# Autotaxin Overexpression Causes Embryonic Lethality and Vascular Defects

**DOI:** 10.1371/journal.pone.0126734

**Published:** 2015-05-19

**Authors:** Hiroshi Yukiura, Kuniyuki Kano, Ryoji Kise, Asuka Inoue, Junken Aoki

**Affiliations:** 1 Graduate School of Pharmaceutical Sciences, Tohoku University, 6–3, Aoba, Aramaki, Aoba-ku, Sendai, 980–8578, Japan; 2 PREST, Japan Science and Technology Agency, Kawaguchi, Saitama, Japan; 3 CREST, Japan Science and Technology Agency, Tokyo, Japan; The University of Tennessee Health Science Center, UNITED STATES

## Abstract

Autotaxin (ATX) is a secretory protein, which converts lysophospholipids to lysophosphatidic acid (LPA), and is essential for embryonic vascular formation. ATX is abundantly detected in various biological fluids and its level is elevated in some pathophysiological conditions. However, the roles of elevated ATX levels remain to be elucidated. In this study, we generated conditional transgenic (Tg) mice overexpressing ATX and examined the effects of excess LPA signalling. We found that ATX overexpression in the embryonic period caused severe vascular defects and was lethal around E9.5. ATX was conditionally overexpressed in the neonatal period using the Cre/loxP system, which resulted in a marked increase in the plasma LPA level. This resulted in retinal vascular defects including abnormal vascular plexus and increased vascular regression. Our findings indicate that the ATX level must be carefully regulated to ensure coordinated vascular formation

## Introduction

Autotaxin (ATX) is a motogen-like phosphodiesterase that was originally isolated from conditioned medium of human melanoma cells [1]. Previously, ATX was shown to have lysophospholipase D (lysoPLD) activity, which converts lysophosphatidylcholine (LPC) to a bioactive lysophospholipid, lysophosphatidic acid (LPA) [2, 3]. LPA is a lipid mediator with diverse biological functions *in vitro* and *in vivo*, most of which are mediated by G protein-coupled receptors (GPCRs) specific to LPA (LPA_1–6_) [4–6]. ATX knockout mice are embryonic lethal around E9.5~10.5 with vascular defects in the yolk sac and embryos [7, 8]. Aberrant neural tube formation is also observed in ATX knockout embryos [9, 10]. ATX catalytic activity must be responsible for these phenotypes because mutated ATX knock-in embryos, in which a single amino acid responsible for the catalytic activity of ATX was modified, are embryonic lethal [11].

High levels of ATX are found in various biological fluids, such as serum, urine and peritoneal fluid [12]. ATX concentration was increased in fluids from patients with various diseases such as chronic hepatitis [13], follicular lymphoma [14] and some cancers including breast, ovary and pancreas [15–17]. In addition, serum ATX levels from pregnant women were found to be high [18]. Elevated ATXs in some cancers are proposed to contribute to the invasion of cancer cells because ATX promotes proliferation and migration of cancer cells through production of LPA and sequential activation of LPA receptors. However, the roles of elevated ATX levels in other situations such as in the development of the vasculature, is unclear.

Here, we showed that overexpression of ATX caused embryonic lethality with vascular defects, growth retardation and prevented closure of the neural tube. In addition, overexpression of ATX in neonatal period results in a delay in retinal vascularization and a decrease in vessel branching. These results indicates that excess of ATX-LPA signalling induces severe vascular defects, which may induce multiple diseases including cancer.

## Material and Methods

### Reagents and antibodies

1-Myristoyl (14:0)-LPC was purchased from Avanti Polar Lipids Inc. Rat anti-CD31 antibody was purchased from BD Biosciences. Biotinylated *Griffonea Simplicifolia* I isolectin B4 was purchased from vector laboratories. Rabbit anti-mouse collagen IV was purchased from AbD Serotec. Alexa Fluor 488 Goat anti-rat IgG, Alexa Fluor 568 goat anti-rabbit IgG and Alexa Fluor 488 streptavidin were purchased from Molecular Probes.

### Mice

**Fig 1:**
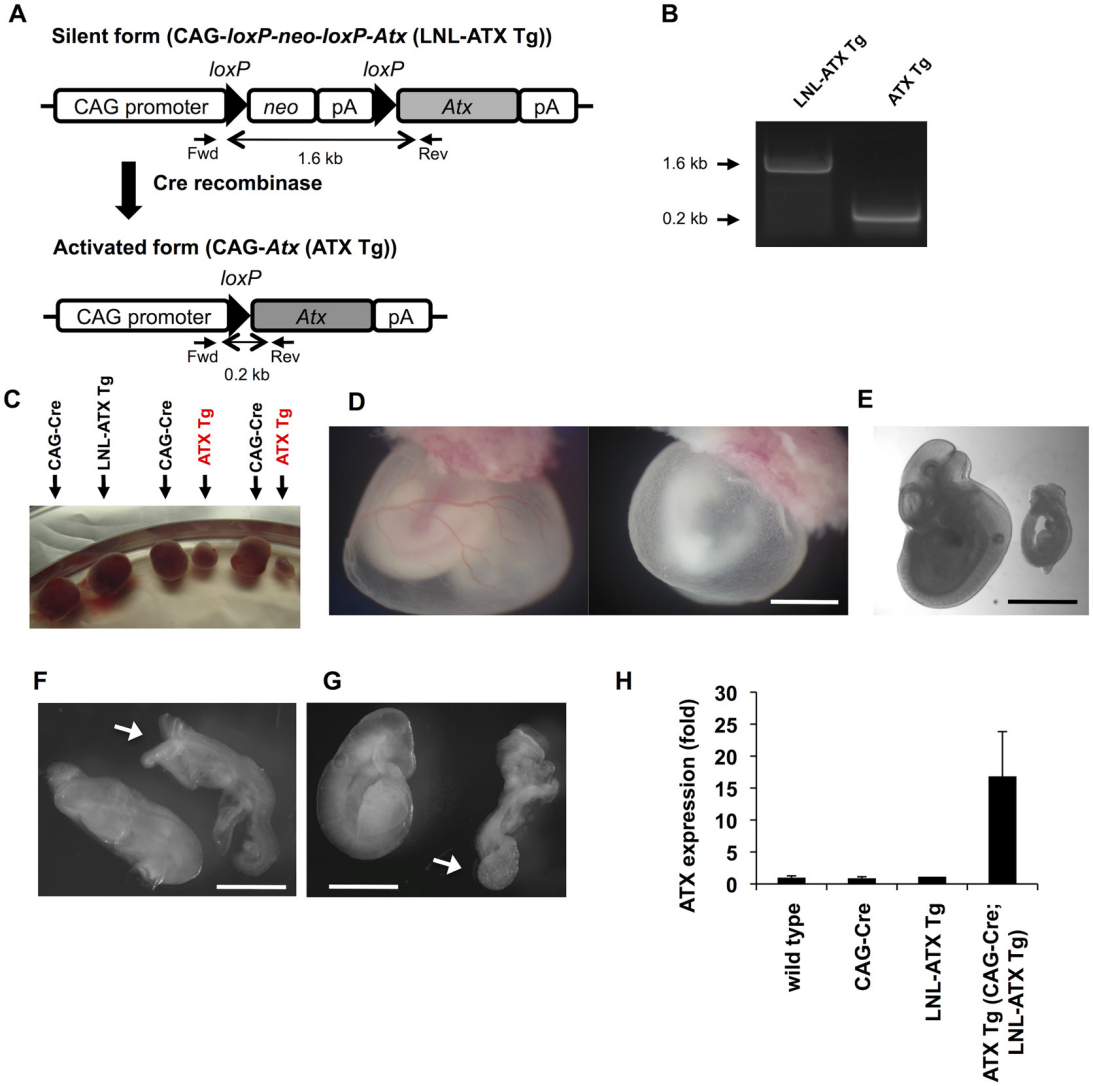
Overexpression of ATX in embryos led to lethality with severe defects. (A) Schematic diagram of the construction of ATX Tg mice. The ATX transgene was inserted at the downstream of the neo^r^/pA cassette. This fragment, in which the ATX transgene is silent, was introduced into mice, and the transgene-positive offspring were then mated with CAG-Cre Tg mice. At this stage, the LNL (for loxP-neo^r^/pA-loxP) cassette was excised by Cre recombinase, and the ATX transgene was activated under control of the CAG promoter in the transgene-positive embryos. (B) PCR genotyping of ATX Tg mice. After mating of LNL-ATX Tg mice with CAG-Cre Tg mice, PCR genotyping was performed. Fragments of 1.6 and 0.2 kb were amplified for LNL-ATX Tg and CAG-ATX Tg (ATX Tg) mice, respectively, whereas these products were not detected in WT littermates. (C) A picture of embryos and placentas at E11.5. (D) Defects in the yolk sac vasculature. Yolk sac from control (wild type) and ATX Tg embryos at E10.5. (E-G) Morphologies of control (wild type) (left) and ATX Tg (right) embryo proper at E9.5 and E10.5. At E10.5 (E) and E9.5 (F and G), ATX Tg embryos are easily distinguishable from control littermates (wild type). ATX Tg embryos exhibit several defects such as growth retardation (E), open and kinky neural tube (F, arrow) and abnormal allantois (G, arrow). Scale bars, 200 μm in panels D and E and 100 μm in panels F and G. (H) Quantitative RT-PCR analysis of ATX mRNA in mouse embryos at E8.5. (wild type; n = 3, CAG-Cre; n = 3, LNL-ATX Tg; n = 2, ATX Tg; n = 3,).

Mice were maintained according to the Guidelines for Animal Experimentation of Tohoku University and the protocol was approved by the Institutional Animal Care and Use Committee at Tohoku University. The strategy for the generation of ATX Tg mice has been reported previously [19]. In brief, the cDNA for mouse ATX (ATXβ isoform) was inserted into the pCALNL5 vector [20]. The plasmid, containing the transgene downstream of a neomycin cassette with LoxP sites at both ends, was excised to produce a CAG-loxP-neo^r^-loxP-ATX (LNL-ATX) fragment. The fragment was then microinjected into fertilized eggs, and the eggs were transferred to the fimbriae of the uterine tubes of female C57BL/6 mice that had been mated with vasoligated male mice 1 day before. Founders were mated with C57BL/6 mice to confirm germ line transmission by PCR genotyping, and those with successful germ line transmission (LNL-ATX Tg mice) were then crossed with CAG-Cre Tg mice. This step resulted in removal of the neo^r^ cassette from the LNL-ATX transgene, thereby allowing activation of the ATX transgene in the whole body of the offspring (Fig 1). To obtain ATX conditional Tg (ATX cTg) mice, LNL-ATX Tg mice were crossed with mice expressing tamoxifen-inducible Cre recombinase (Cre-ER) under the control of the CAG promoter (Jackson Laboratories). Gene activation in pups was triggered by *i*.*p*. injection of 50 μl of tamoxifen solution (Sigma, T5648; 3 mg/ml corn oil) once at P1 or P5. The phenotypes of the mutant mice were analyzed at P6. Other littermates were used as controls. Primers used in genotyping are listed below:

Fwd, 5’-CTTTTTCCTACAGCTCCTGGG-3’ and Rev, 5’-CCATTCGGCCCTCTTAATTCG-3’

### Quantitative RT-PCR analysis

Total RNA from mouse embryos were isolated using a GenElute Mammalian Total RNA Miniprep Kit (Sigma-Aldrich). Total RNA was reverse-transcribed using High-Capacity cDNA RT Kits (Applied Biosystems) according to the manufacturer’s instructions. PCR reactions were performed with SYBR Premix Ex Taq (Takara Bio) and were monitored by ABI Prism 7300 (Applied Biosystems). Standard plasmids ranging from 10^2^ to 10^6^ copies per well were used to quantify the absolute number of transcripts of cDNA samples. The numbers of transcripts were normalized to the number of a house-keeping gene, *Gapdh* in the same sample. Primers used in gene expressions are listed below:

Atx, 5’-GGAGAATCACACTGGGTAGATGATG-3’ and 5’-ACGGAGGGCGGACAAAC-3’;

Gapdh, 5’-AGGAGCGAGACCCCACTAAC-3’ and 5’-CGGAGATGATGACCCTTTTG-3’.

### Measurement of lysophospholipase D activity and Western blotting

Lysophospholipase D activity was measured as described previously [2]. Briefly, plasma samples were mixed with 14:0 LPC (100 mM Tris-HCl, 5 mM MgCl2, 500 mM NaCl, 0.05% Triton X-100, pH 9.0) and incubated for 3 h at 37°C. Liberated choline was quantified using choline oxidase (Wako, Osaka, Japan), peroxidase (TOYOBO, Osaka, Japan) and TOOS reagent (Dojindo, Kumamoto, Japan). The activity was indicated by the generation rate of choline per unit time and volume (pmol/ml/h). Western blotting of ATX was performed as described using ATX-specific monoclonal antibody [7].

### Whole-mount staining and immunofluorescence staining

For immunostaining of flat-mount retinas, eyes were dissected from neonatal mice and fixed in 4% PFA for 2 hrs at room temperature. The retinas were stained with isolectin B4 (1:50), anti-CD31 antibody (1:200) and anti-collagen IV antibody (1:200) as previously described [21].

### Quantification of LPA by LC-MS/MS

Plasma LPA levels were determined by LC-MS/MS as previously described [22]. Briefly, lipids in plasma were extracted in 100 μL of methanol containing 100 nM 17:0-LPA (internal standard). After filtration through a 0.2 μm acetyl cellulose filter (YMC), 20 μL of sample was injected into liquid chromatography (LC) and analyzed by tandem mass spectrometry (MS/MS).

## Results and Discussion

### Overexpression of ATX in embryos led to lethality with severe defects

To obtain transgenic (Tg) mice overexpressing ATX, a Tg construct for ATX (Fig 1A) was microinjected into the pronuclei of fertilized eggs of C57BL/6 females and transferred into the oviducts of pseudopregnant females. The founder mouse, in which the ATX transgene was still silent, was mated with CAG-Cre Tg mice to allow the removal of the neo^r^ cassette from the LNL-ATX transgene by the Cre/LoxP reaction (Fig 1A and 1B). CAG-Cre Tg mice is useful to induce Cre-mediated recombination all tissues because CAG promoter directs ubiquitous expression of the gene [23]. We established three lines, which possessed the ATX Tg allele. Among the three lines, two lines (line D and F) were crossed with CAG-Cre mice. Following this, the transcription of the ATX transgene was directly regulated by the CAG promoter and was thereby activated in all tissues CAG-Cre mice and LNL-ATX Tg mice were healthy and fertile. However, no offspring carrying the active ATX transgene (ATX Tg) was found among 73 newborn mice from LNL-ATX Tg females crossed with CAG-Cre males (Table 1 and S1 Table), suggesting that the ATX Tg mice are embryonic lethal.

**Table 1:** Genotype distribution of offspring from LNL-ATX Tg females crossed with CAG-Cre males.

Number of litters	Total number of neonates tested	Wild type	LNL-ATX Tg	CAG-Cre	CAG-Cre; LNL-ATX Tg
12	73	22	25	26	0

To know when ATX Tg embryos showed abnormality, embryos were isolated at various stages of gestation. At E11.5 almost all of ATX Tg embryos were dead in utero (Fig 1C). At E10.5, the yolk sacs of ATX Tg embryos completely lacked large vitelline vessels, whereas yolk sacs of control (wild type) embryos had well developed vitelline vessels (Fig 1D). In addition, several defects were evident in the ATX Tg embryos at E10.5 or 9.5. These included growth retardation (Fig 1E), open and kinky neural tubes (Fig 1F) and abnormal allantois (Fig 1G). We did not observe any abnormality of embryos at both E9.5 and E10.5 with genotypes of wild type, CAG-Cre and LNL-ATX Tg. The expression levels of ATX mRNA in ATX Tg embryos as judged by RT-PCR were increased about 15-fold compared with those in control embryos at E8.5 (Fig 1H).

### Overexpression of ATX delays retinal vascularization and decreases vessel branching

**Fig 2:**
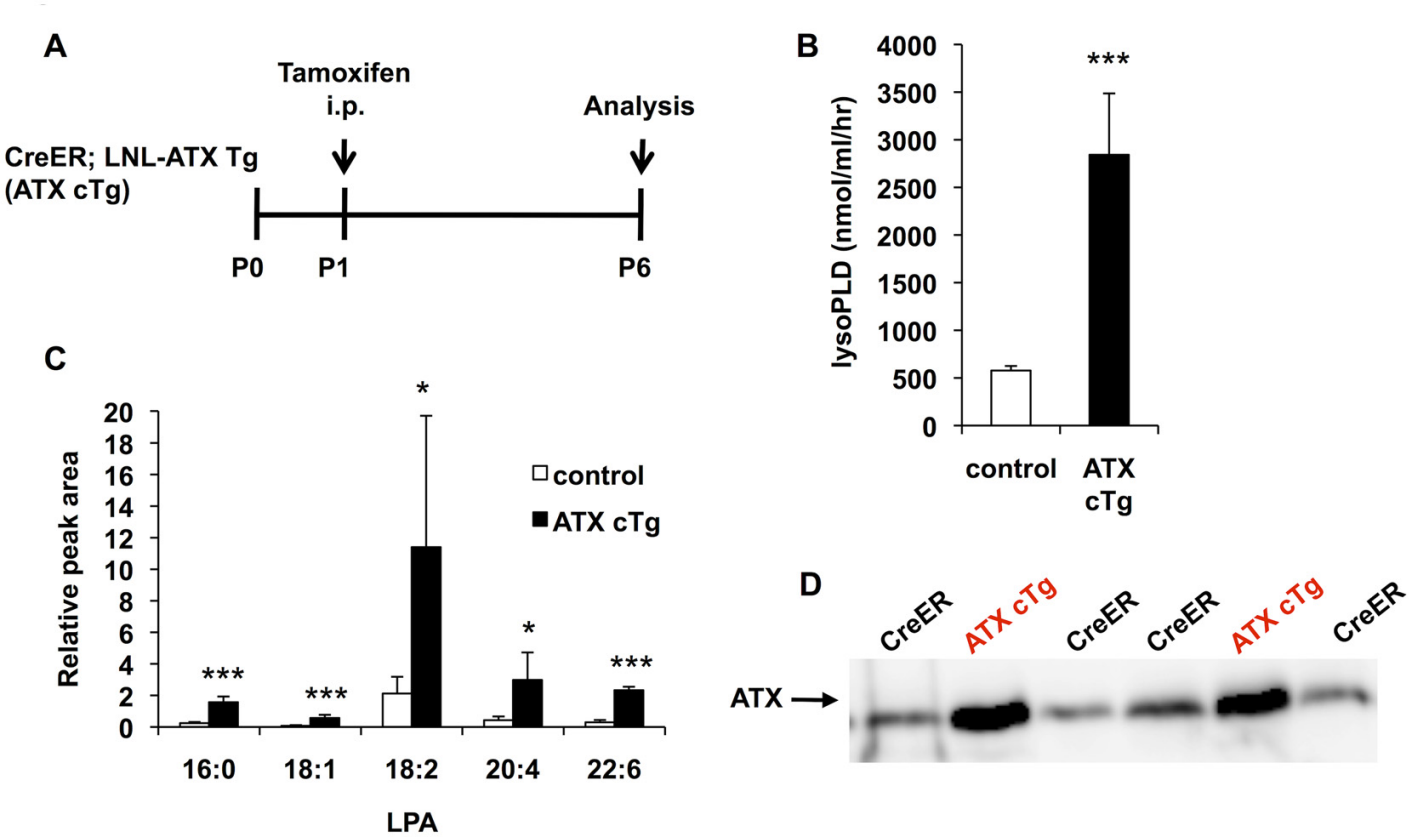
ATX expression is increased in ATX conditional transgenic mice. (A) Schematic of the experimental strategy to assess formation of the retinal vasculature in ATX conditional Tg (ATX cTg) mice. (B) LysoPLD activity of ATX cTg mice plasma. LysoPLD activity was determined by liberation of choline from lysophosphatidylcholine (LPC) using 14:0-LPC as a substrate. Error bars indicate *s*.*d*. (control; n = 12, ATX cTg; n = 7). P-values were estimated by student’s *t*-test, ***P < 0.001. (C) Relative abundance of five major LPA species (16:0, 18:1, 18:2, 20:4 and 22:6-LPA) in mice plasma. Lipids in plasma were extracted with methanol and analyzed by LC-MS/MS. Error bars indicate *s*.*d*. (control; n = 12, ATX cTg; n = 7). P-values were estimated by one-way ANOVA with Bonferroni’s posttest analyses, *P < 0.05, ***P < 0.001. (D) Western blot analysis of ATX in plasma isolated from CreER and ATX cTg mice. Data in (B) and (C) were pooled from three independent experiments.

Next, to assess the effect of ATX overexpression on the vasculature more precisely, we conditionally overexpressed ATX (ATX conditional Tg (cTg) mice) just after birth and evaluated blood vessel formation in neonatal retina [21, 24]. To obtain ATX cTg mice, LNL-ATX Tg mice were crossed with mice expressing tamoxifen (TM)-inducible Cre recombinase (Cre-ER) under the control of the CAG promoter. Gene activation in neonates was triggered by *i*.*p*. injection of tamoxifen solution once at postnatal day 1 (P1). The phenotypes of the ATX cTg mice were analyzed at P6 (Fig 2A). ATX gene activation in ATX cTg mice significantly increased LysoPLD activity (Fig 2B), plasma LPA levels (Fig 2C) and ATX protein level (Fig 2D) compared with the levels in control mice.

**Fig 3:**
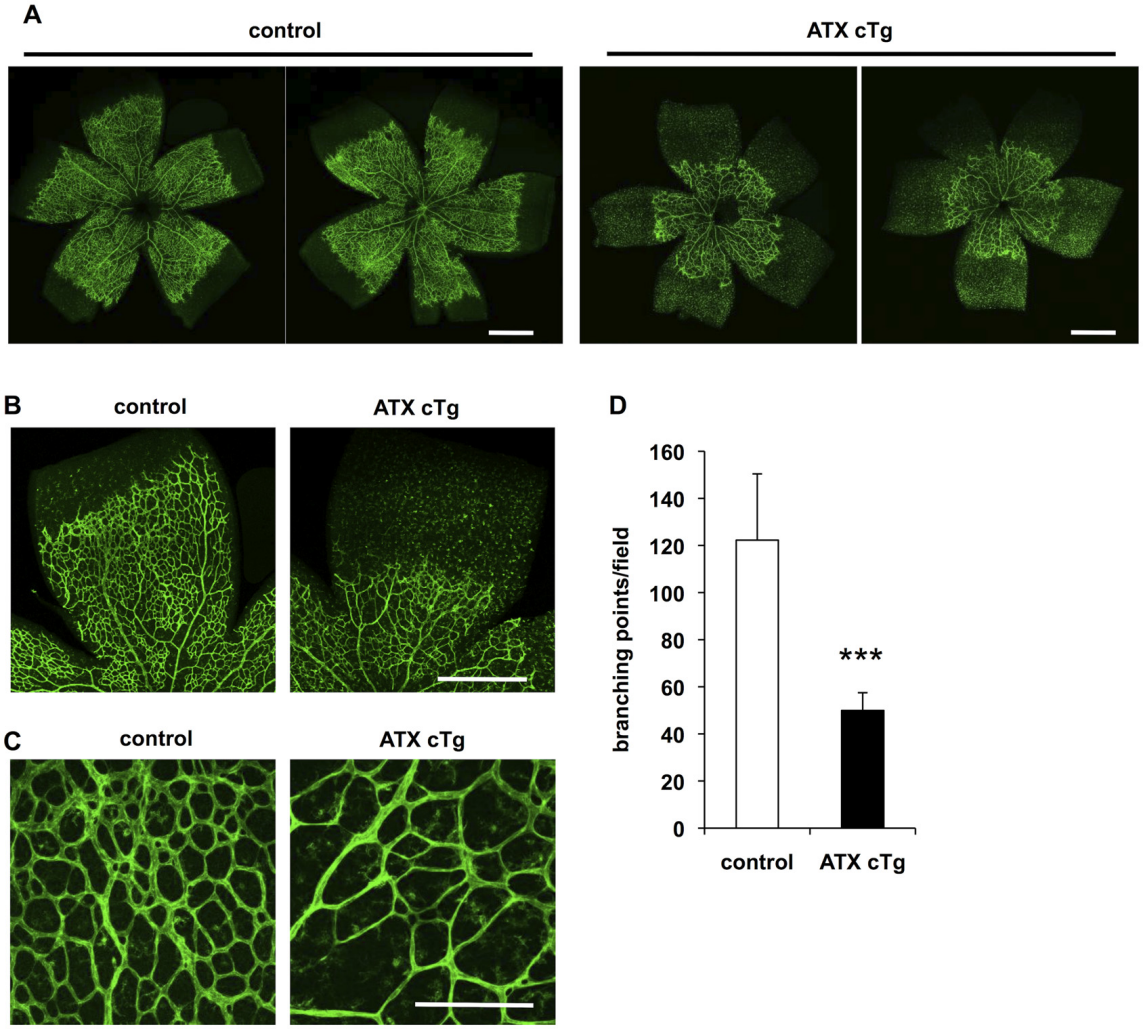
Overexpression of ATX delays retinal vascularization and decreases vessel branching. (A and B) Vascular defects in retina from ATX cTg mice at P6. Retina vasculature was visualized by staining the vessels with isolectin B4. Scale bar, 500 μm. (C) Magnification view of vascular plexus in retina from ATX cTg mice at P6. Scale bar, 100 μm. (D) The vascular defects were evaluated by determining the branching points quantitatively. Error bars indicate *s*.*d*. (control; n = 12, ATX cTg; n = 7). P-values were estimated by student’s *t*-test, ***P < 0.001. Data were pooled from three independent experiments.

ATX cTg mice also displayed a significant delay in radial expansion of vascular plexus from the optic nerve head to the periphery in retina (Fig 3A and 3B) and decreased vascular density and branching (Fig 3C and 3D). We did not observe any differences of retinal vasculature between the three genotypes (wild type, CAG-CreER and LNL-ATX Tg). Therefore, we used neonates with a wild type genotype as a control. Phenotype of delay in radial expansion of vascular plexus and decreaed vascular branching was mainly caused by abnormality of angiogenic sprouting [25, 26], vascular instability [27, 28].

### Transient ATX overexpression decreased vessel branching but did not delay retinal vascularization

**Fig 4:**
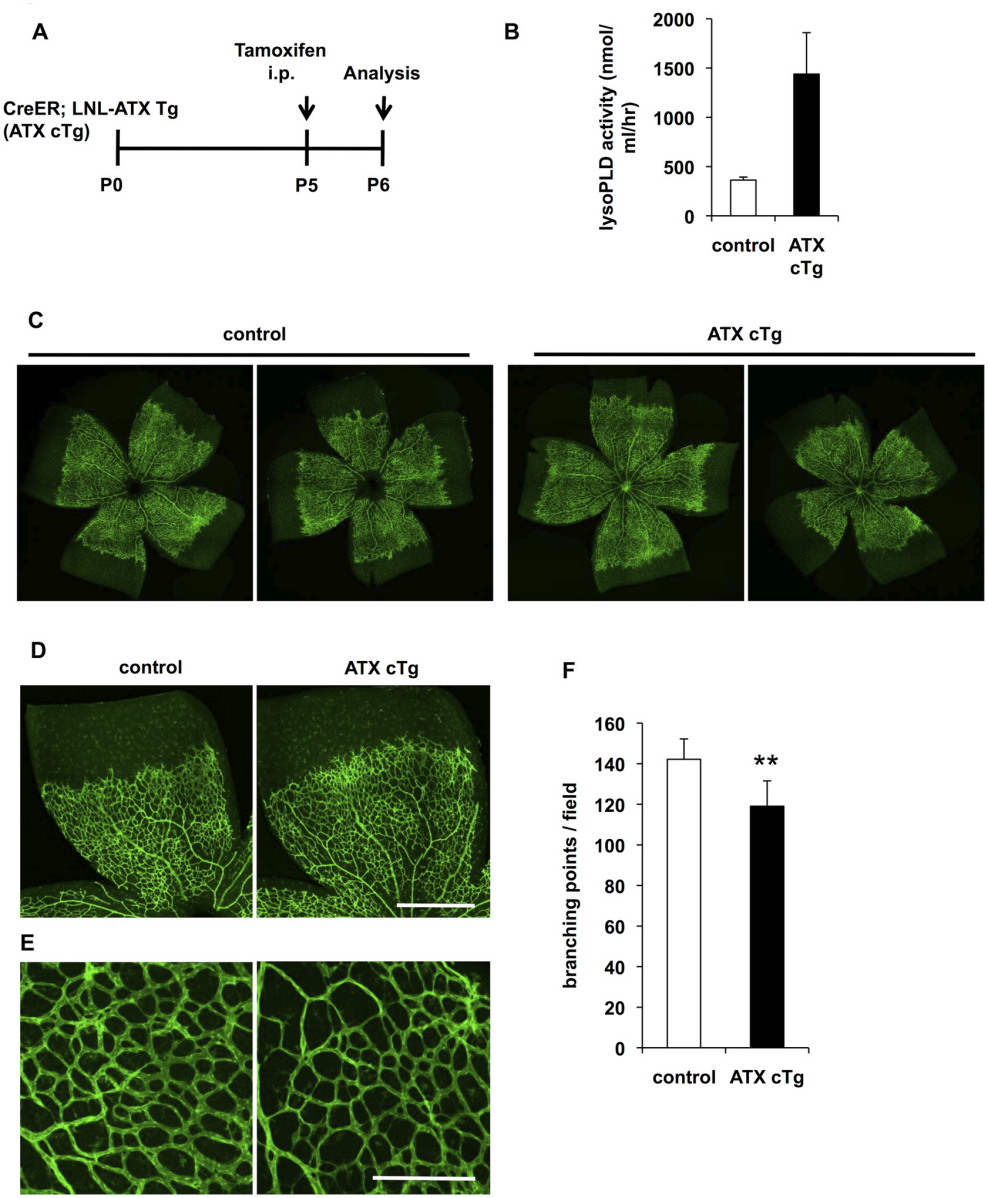
Transient ATX overexpression decreases vessel branching but does not delay retinal vascularization. (A) Schematic of the experimental strategy to assess initial defects in retinal vasculature in ATX cTg mice. (B) LysoPLD activity of ATX cTg mice plasma. Error bars indicate *s*.*d*. (control; n = 9, ATX cTg; n = 4). (C and D) Vascular defects in retina from ATX cTg mice at P6. Retina vasculature was visualized by staining the vessels with isolectin B4. Scale bar, 500 μm. (E) Magnification view of vascular plexus in retina from ATX cTg mice at P6. Scale bar, 100 μm. (F) The vascular defects were evaluated by determining the branching points quantitatively. Error bars indicate *s*.*d*. (control; n = 9, ATX cTg; n = 4). P-values were estimated by student’s *t*-test, **P < 0.01. Data in (B) and (F) were pooled from three independent experiments.

To confirm the effects of ATX overexpression against vascular formation, we induced ATX overexpression at P5 and analyzed the effects at P6 (Fig 4A). Under this condition, plasma LysoPLD activities in ATX cTg mice increased about 4-fold compared with those in control mice (Fig 4B). In ATX cTg mice, radial expansion of the vascular plexus was not delayed (Fig 4C and 4D) but vascular branching was slightly decreased (Fig 4E and 4F). These results indicate that LPA causes vascular instability rather than prevents vascular sprouting.

### Overexpression of ATX causes abnormal vessel morphology and vessel regression

**Fig 5:**
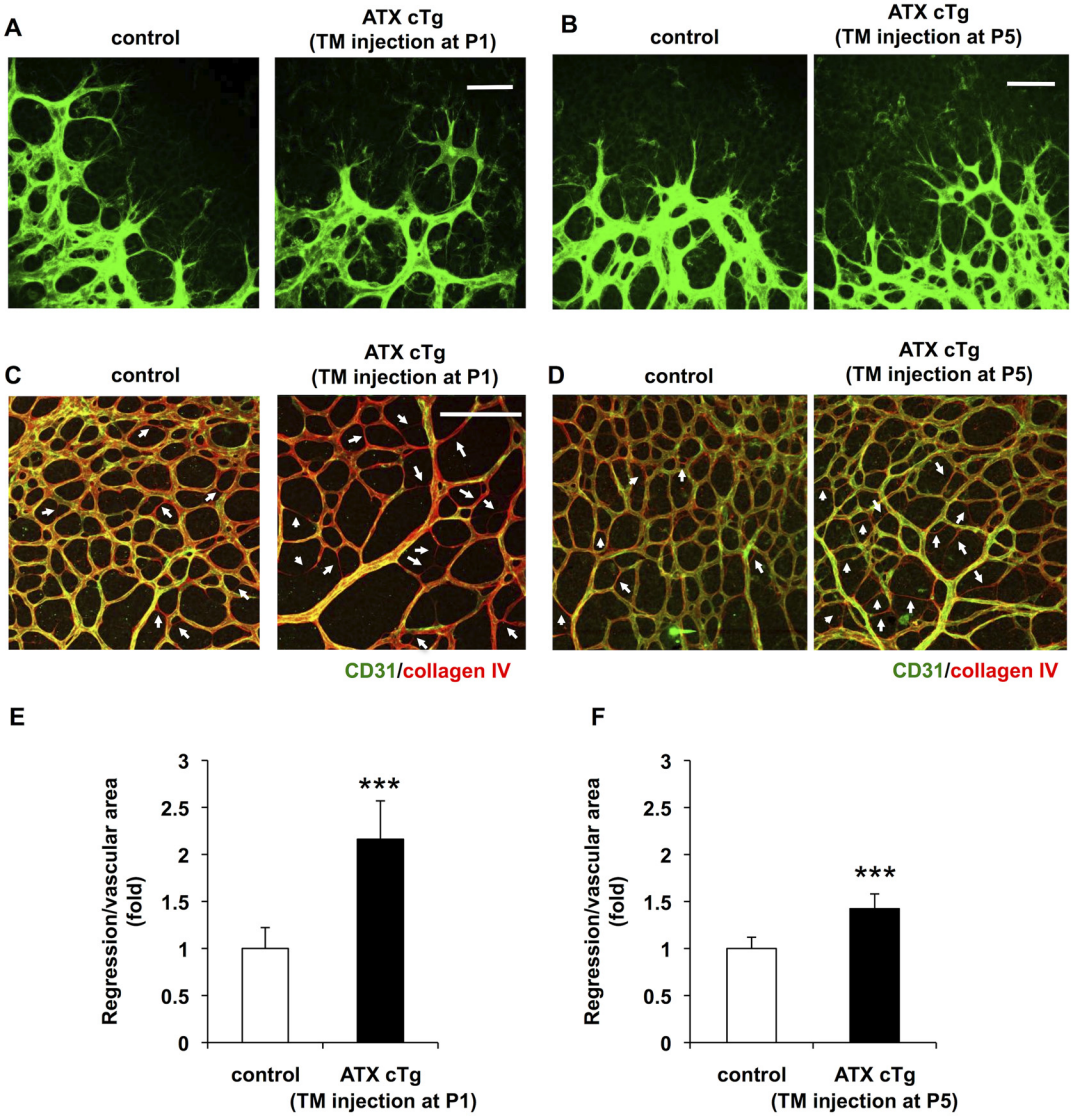
Overexpression of ATX causes abnormal vessel morphology and vessel regression. (A and B) Magnification view of angiogenic front in retina from ATX cTg mice at P6. Control and ATX cTg retinas had similar filopodia protrusion. Scale bar, 50 μm. TM, tamoxifen. (C and D) ATX cTg retinas displayed vessel regression at vascular plexus. Control (wild type) and ATX cTg retinas labeled for CD31 (green) and collagen IV (red). Arrows highlight empty collagen IV sleeves, indicating vessel regression. Scale bar, 100 μm. (E and F) Vessel regression was evaluated quantitatively. Error bars indicate *s*.*d*. (control; n = 7, ATX cTg; n = 5). P-values were estimated by student’s *t*-test, ***P < 0.001. Data were pooled from three independent experiments.

At higher magnification, the formation of filopodia in both ATX cTg mice (TM injection at P1 or P5) was not affected (Fig 5A and 5B), suggesting that sprouting activity was not suppressed by overexpressing of ATX. When retinas were stained with both anti-CD31 (an endothelial marker) and anti-collagen IV (basement membrane marker), we found many vessels those were positive only for collagen IV, namely endothelial cell-deficient vessels, in ATX cTg retinas (Fig 5C–5F). The presence of endothelial cell-deficient (empty) vessels in ATX cTg retinas indicates that endothelial cells regressed after basement membranes were formed around the endothelial cells, as was demonstrated previously in VEGF inhibited vessels [29]. The observed vascular regression could explain the delayed retinal vascularization and the reduced vascular density in ATX cTg retinas because previous studies showed vessel instability (regression) led to retardation of retinal vascularization and decreased vessel branching [27, 28].

In this study, we found that overexpression of ATX caused embryonic lethality with vascular defects, growth retardation and failure to close the neural tube. In addition, overexpression of ATX in the neonatal period caused vascular instability thereby inducing a delay in retinal vascularization and a decrease in vessel branching. By evaluating developing retinal vasculature in detail, we also found excess ATX-LPA signaling induced vessel regression and defects of vascular elongation (Figs 3 and 5), as was previously reported by Im et al., who showed that ATX-LPA signaling induced hyaloid vessel regression [30].

Because knocking out ATX in mice and down-regulating ATX in zebrafish severely inhibited embryonic blood vessel formation [7, 8, 31], ATX as well as its product LPA has been thought to be an essential angiogenic factor. However, the present results show that excess ATX-LPA signalling induces severe vascular defects and thus indicate that excess LPA signalling inhibits angiogenesis. This indicates that the LPA level must be regulated tightly. Lipid phosphate phosphatase 3 (LPP3) is a candidate enzyme for LPA degradation. Interestingly, LPP3 knockout mice were also embryonic lethal with vascular defects similar to those of ATX-Tg mice [32]. It would be interesting to know whether LPP3 knockout elevates the LPA level and strengthens LPA signaling. In addition, because ATX levels are high in patho-physiological states such as cirrhosis, pruritus, pregnancy and various cancers [15, 33–35], it would be interesting to know if the increased ATX level affects blood vessel formation in clinical conditions.

## Supporting Information

S1 Table Genotype distribution of offspring from LNL-ATX Tg females (D and F line) crossed with CAG-Cre males. (DOCX)
