# Supplementary material for: Autotaxin Overexpression Causes Embryonic Lethality and Vascular Defects
Source: PLoS One. 2015 May 19;10(5):e0126734. doi: 10.1371/journal.pone.0126734 (PMC4438000; doi:10.1371/journal.pone.0126734)
Supplement: S1 Table — (DOCX) [file pone.0126734.s001.docx]

**Table S1.**

**Genotype distribution of offspring from LNL-ATX Tg females (D and F line) crossed with CAG-Cre males.**

| Tg line | Number of litters | Total number of neonates tested | Numbers of neonates | | | |
| --- | --- | --- | --- | --- | --- | --- |
|  |  |  | Wild type | LNL-ATX Tg | CAG-Cre | CAG-Cre; LNL-ATX Tg |
| D line | 6 | 37 | 11 | 14 | 12 | 0 |
| F line | 6 | 36 | 11 | 11 | 14 | 0 |
